# Physician vs. AI-generated messages in urology: evaluation of accuracy, completeness, and preference by patients and physicians

**DOI:** 10.1007/s00345-024-05399-y

**Published:** 2024-12-27

**Authors:** Eric J. Robinson, Chunyuan Qiu, Stuart Sands, Mohammad Khan, Shivang Vora, Kenichiro Oshima, Khang Nguyen, L. Andrew DiFronzo, David Rhew, Mark I. Feng

**Affiliations:** 1 Department of Urology, Los Angeles Medical Center, Kaiser Permanente, Los Angeles, CA USA; 2 Department of Anesthesiology, Baldwin Park Medical Center, Kaiser Permanente, Baldwin Park, CA USA; 3 Kaiser Permanente, Pleasanton, CA USA; 4 Microsoft Health & Life Sciences, Irvine, CA USA; 5 Microsoft Health & Life Sciences, Dallas, TX USA; 6 Kaiser Permanente, Oakland, CA USA; 7 Department of Family Medicine, Kaiser Permanente, Pasadena, CA USA; 8 Kaiser Permanente, Pasadena, CA USA; 9 Microsoft Health & Life Sciences, Redmond, WA USA; 10 Department of Urology, Baldwin Park Medical Center, Kaiser Permanente, 1011 Baldwin Park Blvd., Baldwin Park, CA 91706 USA

**Keywords:** Artificial intelligence (AI), Large language models (LLMs), Benign prostatic hyperplasia (BPH), Patient communication, Patient messages, Sandbox, ChatGPT, Chatbot, Care experience, Physician experience

## Abstract

**Purpose:**

To evaluate the accuracy, comprehensiveness, empathetic tone, and patient preference for AI and urologist responses to patient messages concerning common BPH questions across phases of care.

**Methods:**

Cross-sectional study evaluating responses to 20 BPH-related questions generated by 2 AI chatbots and 4 urologists in a simulated clinical messaging environment without direct patient interaction. Accuracy, completeness, and empathetic tone of responses assessed by experts using Likert scales, and preferences and perceptions of authorship (chatbot vs. human) rated by non-medical evaluators.

**Results:**

Five non-medical volunteers independently evaluated, ranked, and inferred the source for 120 responses (*n* = 600 total). For volunteer evaluations, the mean (SD) score of chatbots, 3.0 (1.4) (moderately empathetic) was significantly higher than urologists, 2.1 (1.1) (slightly empathetic) (*p* < 0.001); mean (SD) and preference ranking for chatbots, 2.6 (1.6), was significantly higher than urologist ranking, 3.9 (1.6) (*p* < 0.001). Two subject matter experts (SMEs) independently evaluated 120 responses each (answers to 20 questions from 4 urologist and 2 chatbots, *n* = 240 total). For SME evaluations, mean (SD) accuracy score for chatbots was 4.5 (1.1) (nearly all correct) and not significantly different than urologists, 4.6 (1.2). The mean (SD) completeness score for chatbots was 2.4 (0.8) (comprehensive), significantly higher than urologists, 1.6 (0.6) (adequate) (*p* < 0.001).

**Conclusion:**

Answers to patient BPH messages generated by chatbots were evaluated by experts as equally accurate and more complete than urologist answers. Non-medical volunteers preferred chatbot-generated messages and considered them more empathetic compared to answers generated by urologists.

**Supplementary Information:**

The online version contains supplementary material available at 10.1007/s00345-024-05399-y.

## Introduction

There is growing integration of artificial intelligence (AI) into healthcare, particularly with Large Language Models (LLMs). LLMs leverage self-supervised learning to acquire knowledge from extensive unannotated datasets [1, 2]. Chatbots, implementations of LLMs defined as an AI software capable of engaging in conversational exchanges, are increasingly considered as tools for improving physician-patient communication [3, 4]. A clinically applicable chatbot can serve physicians and patients through real-time actionable information exchanges and delivering on physician objectives for quality and personalized care.

ChatGPT, a widely popular chatbot with broad training on internet sources including medical books and articles, has demonstrated an ability to discuss and answer medical questions with a high degree of complexity and clarity [5, 6]. The availability and usability of ChatGPT make it a popular consult for patients seeking information on healthcare symptomatology and treatment, and patients are trending toward using chatbots more than online search engines [7, 8]. However, in clinical practice, the use of individualized data outside of a hospital’s technical ecosystem creates ethical and legal liabilities as LLMs store and train on input data, exposing personal health information (PHI) to systems outside of a healthcare institution’s control [9]. Integration with any outside system is a security risk, and effective handling of PHI requires development of systems with structural integrity from data storage to access controls.

A “sandbox” environment is a secure testing space used in developing healthcare technology that replicates a healthcare software ecosystem isolated from patient data. Use of sandboxes eliminates the risk of data breaches while allowing robust testing of new technologies. In our study, we established a sandbox environment at a large, integrated health system to ensure secure testing, selecting Benign Prostatic Hyperplasia (BPH) as an initial test topic.

BPH is a common urologic condition well suited for chatbot testing due to its high prevalence and large message volume. Primarily affecting males over 50, BPH involves a relatively focused demographic that serves as an effective test group to study chatbot effectiveness in clinical communications. Due to appropriate precaution surrounding PHI, existing studies assessing the ability of chatbots to answer medical topics have used questions drawn from social media, FAQs on clinic websites, education guidelines, or generated by physicians [5–7, 10–13]. This approach limits the full range of a chatbot’s ability to respond to open-ended, personalized and patient-specific questions. To our knowledge, the use of chatbots in perioperative settings along a patient’s entire surgical journey, from preoperative evaluation and consent to postoperative follow up and recovery expectations, has not been studied.

By leveraging a sandbox environment, our study uses the most common real-world patient questions as selected by a nursing messaging management team responsible for triaging hundreds of questions per week. This study’s selected questions are open-ended, covering topics across the spectrum of care for a common urologic condition, emulating the complexity and ambiguity that clinicians and patients encounter during the regular course of clinical care. Our study evaluates answers from four board-certified urologists and two chatbots for accuracy, comprehensiveness, tone, and patient preference. By evaluating chatbot performance on a range of common clinical questions, this study provides early evidence for the reliability of chatbots in a real-world clinical setting to generate accurate and complete information in an empathetic manner.

## Methods

This cross-sectional study was exempt from Institutional Review Board review as no PHI were used. This study adhered to the guidelines set forth by the STROBE statement for observational research reporting [14]. Informed consent was not obtained by respondents but was implied by participation in survey response. Respondents and evaluators were not compensated.

Twenty common BPH-related patient questions from phone or EHR-messaging were pooled, anonymized, and compiled by clinic nursing message management team (Supplemental Table 1). A standardized answer key for each of the twenty questions was independently generated by three urologists with subject matter expertise in treating large-volume BPH to serve as a rubric for evaluating accuracy and completeness.

Answers for each question were generated by four urologists and two chatbots. The four urologists were provided a document with the questions and instructed to provide the “best answer possible” as a simulation of answering patient messages. The two chatbots, Kaiser Permanente GPT (KPGPT), a snapshot version of ChatGPT 4.0 LLM (gpt-4-0613), and SurgiChat, the same snapshot of ChatGPT 4.0 with additional Retrieval-Augmented Generation (RAG) based on BPH literature and implemented within a sandbox environment [15, 16]. RAG redirects LLM processing to retrieve relevant information from authoritative, pre-determined knowledge sources. The sandbox was an environment disconnected from the healthcare institution resources and databases to prevent inclusion of PHI/PII sensitive information, and processing was performed at pre-approved regional data centers restricted to institutional IP addresses.

The chatbots were provided each question individually in a new chat session with the prompt *“Be specific and incorporate any applicable applied sources of information”*.

Answers from urologists and chatbots were organized by question and presented in random order to participants. Some chatbot responses qualified answers as non-professional, for example “Consult a healthcare provider to determine the best course of action.” Such qualifications were truncated from the response during answer compilation to maintain blindness to the answer source. The document was distributed to two urologists with subject matter expertise in BPH (SMEs) and five non-medical volunteers pre-screened to be male, 50 years or older, and with at least a high-school level of education. Volunteers aged 56–82 (median 72), three received previous BPH treatment, four had a master’s degree and one associate degree, and four were Caucasian, one was Asian. The SMEs and non-medical volunteers were informed they would be participating in evaluating physician and chatbot answers to common patient questions but were not informed of the identity or number of physicians or chatbots providing answers.

The SMEs were asked to grade each question based on their medical expertise and in accordance with the expert-generated answer key for accuracy, completeness, and tone using Likert scales. Accuracy was graded on a 6-point Likert scale (1, completely incorrect; 2, more incorrect than correct; 3, approximately equal correct and incorrect; 4, more correct than incorrect; 5, nearly all correct; 6, completely correct). Completeness was graded on a 3-point Likert scale (1, incomplete; 2, adequate [addresses all aspects of the question and provides the minimum amount of information to be considered complete]; 3, comprehensive). Feature/tone was graded on a 5-point Likert scale (1, not empathetic; 2, slightly empathetic; 3, moderately empathetic; 4, empathetic; 5, very empathetic).

The non-medical volunteers were asked to grade each answer for feature/tone using the same Likert scale, to rank each answer within each question 1–6 in terms of their preference for receiving a message (1 the answer they would most prefer to receive, and 6 as the answer they would least prefer to receive), and label the answer as generated by human or chatbot.

### Statistical analysis

Score results were listed descriptively and were compared between groups using the Pearson’s Chi-squared test for evaluating response source and Wilcoxon rank sum test or the Kruskal-Wallis test for Likert scales and preference ranking. A 2-sided *P <* 0.05 was considered statistically significant. Interrater agreement between subject matter experts was graded using the weighted κ statistic across all scores for eval (R package “irr”; R, version 4.3.1 [The R Project for Statistical Computing]).

## Results

### Subject matter expert analysis

SMEs each evaluated 120 answers from 4 urologists and 2 chatbots responding to 20 questions each including 7 questions on BPH evaluation, 7 questions on BPH operations, and 6 questions on post-operative recovery, for a total of 240 evaluations (160 from urologists, 80 from chatbots) (Table 1). The median scores across all questions were as follows: urologists had a median accuracy of 5.0 (IQR, 4.0–5.0), completeness of 2.0 (IQR, 1.0–2.0), and a feature/tone of 2.0 (IQR, 2.0–3.0); chatbots had a median accuracy of 5.0 (IQR, 4.0–6.0), completeness of 3.0 (IQR, 2.0–3.0), and feature/tone of 3.0 (IQR, 2.0–4.0). Comparatively, chatbot and urologist responses showed no significant difference in accuracy (mean [SD], 4.5 [1.1] vs. 4.6 [1.2], *p* = 0.2), but chatbots scored higher in completeness (mean [SD], 2.4 [0.8] vs. 1.6 [0.6], *p* < 0.001) and feature/tone (mean [SD], 3.2 [1.3] vs. 2.4 [1.0], *p* < 0.001). Interrater agreement was moderate for accuracy (weighted κ = 0.18; *P* < 0.05) and completeness (weighted κ = 0.34; *P* < 0.001), but low for feature/tone (weighted κ = 0.04; *P* = 0.32).

**Table 1:**
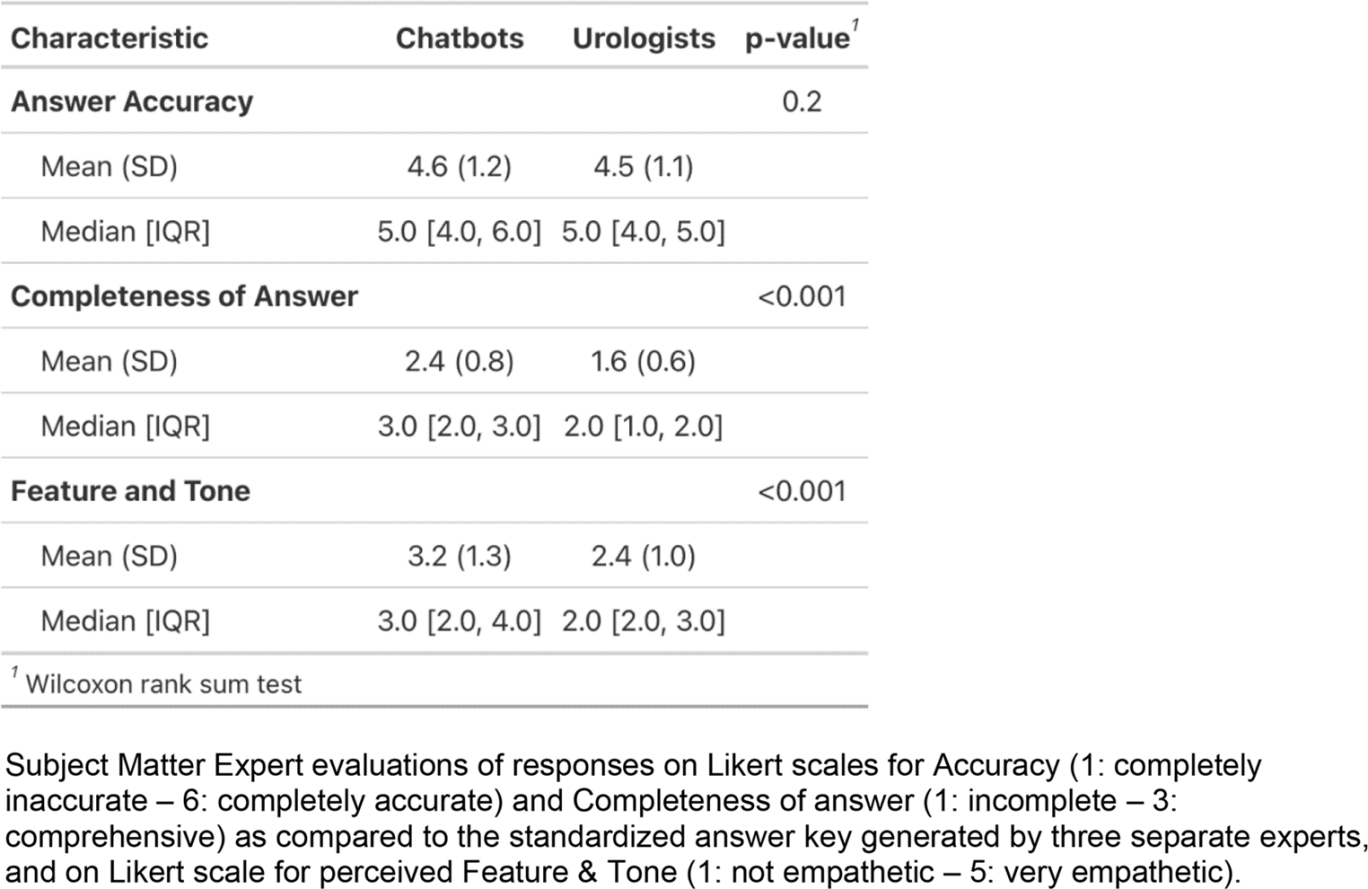
Subject matter expert evaluations

For pre-operative evaluation, chatbots outperformed urologists in accuracy (mean [SD], 5.2 [0.9] vs. 4.4 [1.2], *p* = 0.001), completeness (mean [SD], 2.6 [0.6] vs. 1.6 [0.6], *p* < 0.001), and feature/tone (mean [SD], 2.9 [1.4] vs. 2.1 [1.0], *p* = 0.008) [Supplemental Table 1]. For operative experience, there was no significant difference in accuracy (mean [SD], 4.2 [1.4] vs. 4.8 [1.0], *p* = 0.07) or feature/tone (mean [SD], 3.2 [1.2] vs. 2.7 [1.1], *p* = 0.14) between chatbots and urologists, but chatbots were significantly better in completeness (mean [SD], 2.4 [0.8] vs. 1.6 [0.6], *p* < 0.001). For post-operative experience, no significant difference was found in accuracy (mean [SD], 4.2 [1.1] vs. 4.2 [1.0], *p* > 0.9); however, chatbots scored significantly higher in completeness (mean [SD], 2.0 [0.9] vs. 1.5 [0.6], *p* = 0.007) and feature/tone (mean [SD], 3.5 [1.2] vs. 2.3 [0.8], *p* < 0.001)

### Non-medical volunteer analysis

Non-medical volunteers evaluated 120 responses from 4 urologists and 2 chatbots. Volunteer accuracy in identifying the source was 59% (95% CI: 55.3 − 61.7%), while the expected accuracy of random guessing was 67%. They were not significantly different in identifying chatbot and urologist answers (54% vs. 61%, *p* = 0.089). Median scores for chatbots’ features and tone were higher than for urologists (3.0 [IQR 2.0–4.0] vs. 2.0 [IQR 1.0–3.0]), as were preference rankings (2.0 [IQR 1.0–4.0] vs. 4.0 [IQR 3.0–5.0]). Mean scores confirmed these results (feature/tone: 3.0 [1.4] vs. 2.1 [1.1], preference: 2.6 [1.6] vs. 3.9 [1.6], *p* < 0.001 for both) (Table 2). Scores within categories—evaluation, operative, and post-operative experiences—also favored chatbots, with statistical significance (*p* < 0.005). Responses that volunteers labeled as human scored higher in feature/tone than those labeled as chatbot (2.7 [1.1] vs. 2.1 [1.4], *p* < 0.001), also with a higher preference ranking (3.3 [1.6] vs. 3.8 [1.8], *p* < 0.001) (Supplemental Table 3).

**Table 2:**
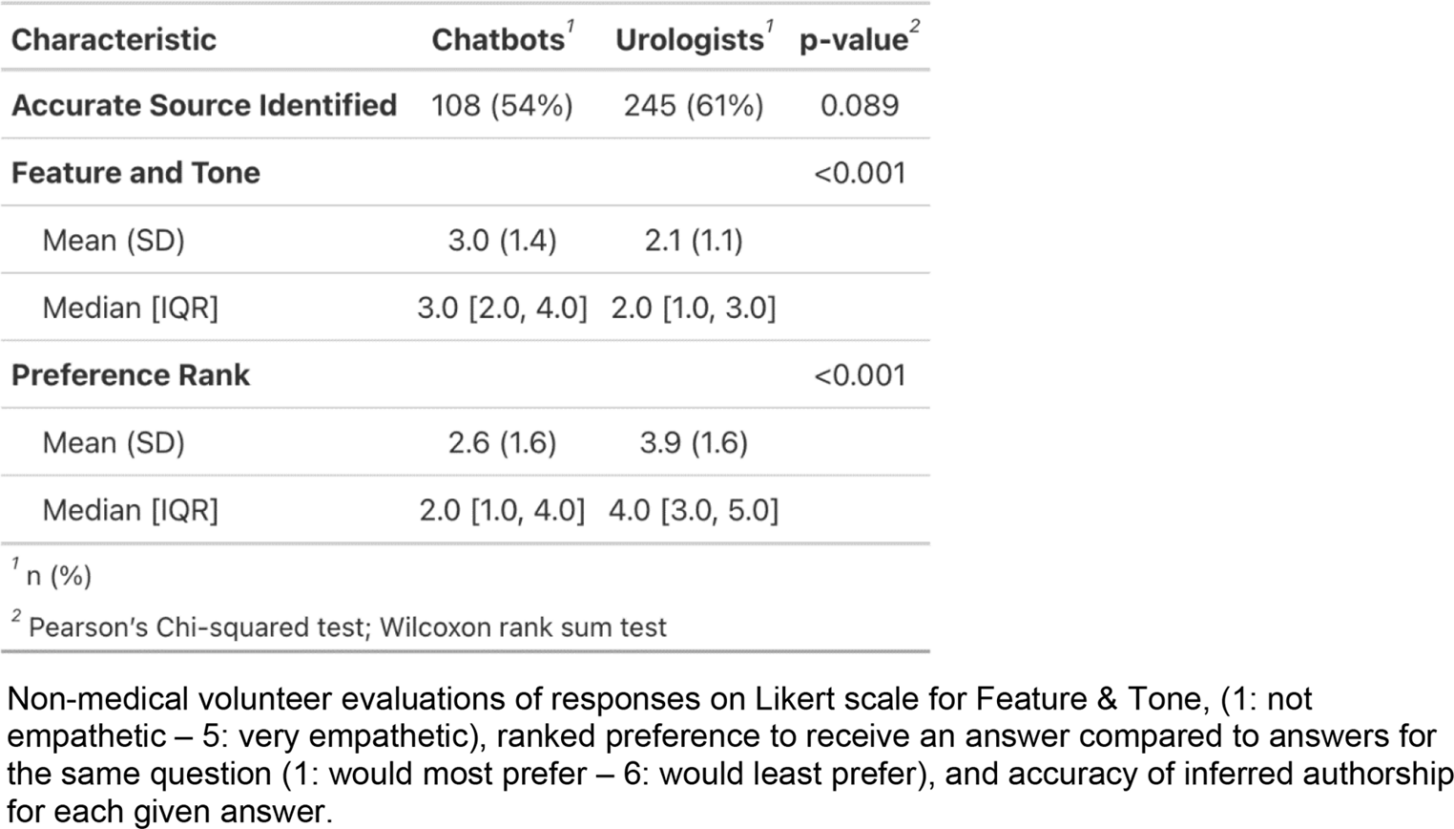
Non-medical volunteer evaluations

## Discussion

In this cross-sectional study, we observed that purpose-trained chatbots and general-purpose chatbots are equally effective in answering patient BPH questions as their expert urologist counterparts. Both chatbots scored better than expert urologists and are preferred by non-medical volunteers when comparing accuracy, completeness, empathy, and readability. Interestingly, non-medical volunteers mistook Chatbot answers for human generated. Furthermore, our purpose-trained chatbot was not better than the general-purpose chatbot in all tested categories.

This study suggests when blinded to authorship, chatbot responses to common BPH questions compared to urologist responses were equally accurate, more complete, more empathetic, and overall preferred by non-medical volunteers. Conversely, this study suggests a negative association with the chatbot label, finding volunteers inferred chatbot authorship for communication they found less desirable and less empathetic.

The use of generative AI in healthcare is growing rapidly, enhancing performance and adoption of the new technology. Our study’s finding that chatbot responses are accurate and complete aligns with prior research in surgical fields that chatbots provide well-structured, comprehensive, and accurate answers to patient medical questions [6, 11, 17, 18]. Chatbot responses in our study maintained high accuracy and coherence with the standardized answer key, with 59% of answers rated a 5/6 or 6/6 accuracy level compared to 53% of urologists. The 9% of chatbot responses with 2/6 accuracy is comparable to the 7% of urologist answers rated 2/6 or lower, and is consistent with ChatGPT accuracy in other studies of general medical messaging [5, 7, 19].

Physicians spend significant time on EHR tasks and patient inquiries, and administrative burden is a primary pain point among urologists, with recent research showing chatbots have potential to reduce administrative burnout by answering patient questions [6, 20]. Typing long answers is time consuming, and it would take 7–8 min to type the median ChatGPT response of 300 words at an average typing speed (40 words per minute) [21]. This may account for the discrepancy in comprehensiveness scores where 55% of chatbot responses were “comprehensive” compared to 5% of urologist responses, and 18% of chatbot responses compared to 53% of urologist responses were “incomplete.” The difference in answer length may account for the difference in completeness, as longer chatbot answers involved defining acronyms and providing broader context with detail outside of the specific question, while urologist answers were more specific and direct.

Given higher empathy evaluations, chatbot implementation offers an opportunity to foster connection with patients through active messaging, a factor associated with improved patient outcomes and satisfaction, without tradeoff for increased feelings of burnout [22–24]. Our volunteers associated more empathetic and preferable messages with human authorship, though the preferred messages were often authored by chatbots. This preference aligns with other studies that patients, especially at lower education levels, view AI messages negatively and express higher trust in physicians than AI-generated information [1, 4, 12, 25]. Urologists are aware of these perceptions, and a 2024 survey of 456 urologists identified negative patient perception as a primary concern of AI-generated information [26]. The concerns around perception and accuracy emphasize the role of urologists as gatekeepers and collaborators in the optimal integration of AI, and the ideal implementation is an active area of research that will individually depend on institutional and regional requirements.

Human processing of messages serves as a guardrail for identifying urgent patient symptoms (e.g. severe hematuria, early sepsis), and the ability of an isolated chatbot to handle such symptoms must be tested, as well as the responsibilities of human oversight in a chatbot-integrated system. Further research and testing of chatbot interactions may cover broader areas of urology, particularly questions with less publicly available information, or areas of higher sensitivity such as erectile dysfunction, or sexually transmitted diseases. ChatGPT has internal guardrails to avoid discussing sensitive or sexual topics, and its consistency in handling sexually explicit questions needs testing. Testing is also required for questions that are irregularly structured (grammatically incorrect, contain multiple incomplete sentences, separate questions or references).

### Limitations

The participants were limited to five non-medical evaluators with above-average education. Further assessment of patients and chatbot messaging should expand the demographic base to include conditions that affect broader ages, genders, non-English speakers, varying levels of education, and types of urologic conditions. Our study did not assess factors that may influence perception of feature & tone or preference, such as patient sentiment or anxiety toward a specific question. There is a positive association between word count with the answer metrics evaluated in this study, but the direct impact of word count was not examined in this study and could be investigated in future research. There was low interrater agreement between SMEs, which may skew the accuracy and completeness findings. Additionally, this study covers a small sample of question prompts that do not perfectly model human speech or text patterns; the questions were limited to a single area of urology that generally pertains to a single gender, contained one or two closely related questions within each prompt, and were written with perfect grammar and spelling. Finally, the summary measures used for evaluation were not validated or pilot tested.

## Conclusion

Chatbot responses to common BPH questions when compared to urologist responses were equally accurate, more complete, more empathetic, and overall preferred by patient volunteers when blinded to the authorship. This study highlights the potential for integration of AI-written messaging into urology office communications. Further studies are needed on the negative perceptions of patient toward AI generated answers.

## Electronic supplementary material

Below is the link to the electronic supplementary material.

Supplementary Material 1

## Data Availability

Data that support the findings of this study are available online in an anonymized format at https://github.com/EricRob/surgichat/blob/main/compiled_evaluations.csv.
