# Supplementary material for: Physician vs. AI-generated messages in urology: evaluation of accuracy, completeness, and preference by patients and physicians
Source: World J Urol. 2024 Dec 27;43(1):48. doi: 10.1007/s00345-024-05399-y (PMC11680670; doi:10.1007/s00345-024-05399-y)

**Supplemental Figures**

**Supplemental Material**

**Supplemental Figure 1: SME ratings of answers, by respondent**

Boxplot of SME evaluations of answers for each respondent with IQR and median, ordered by median word count. Minimum and maximum are omitted from evaluations using Likert scales for clarity.


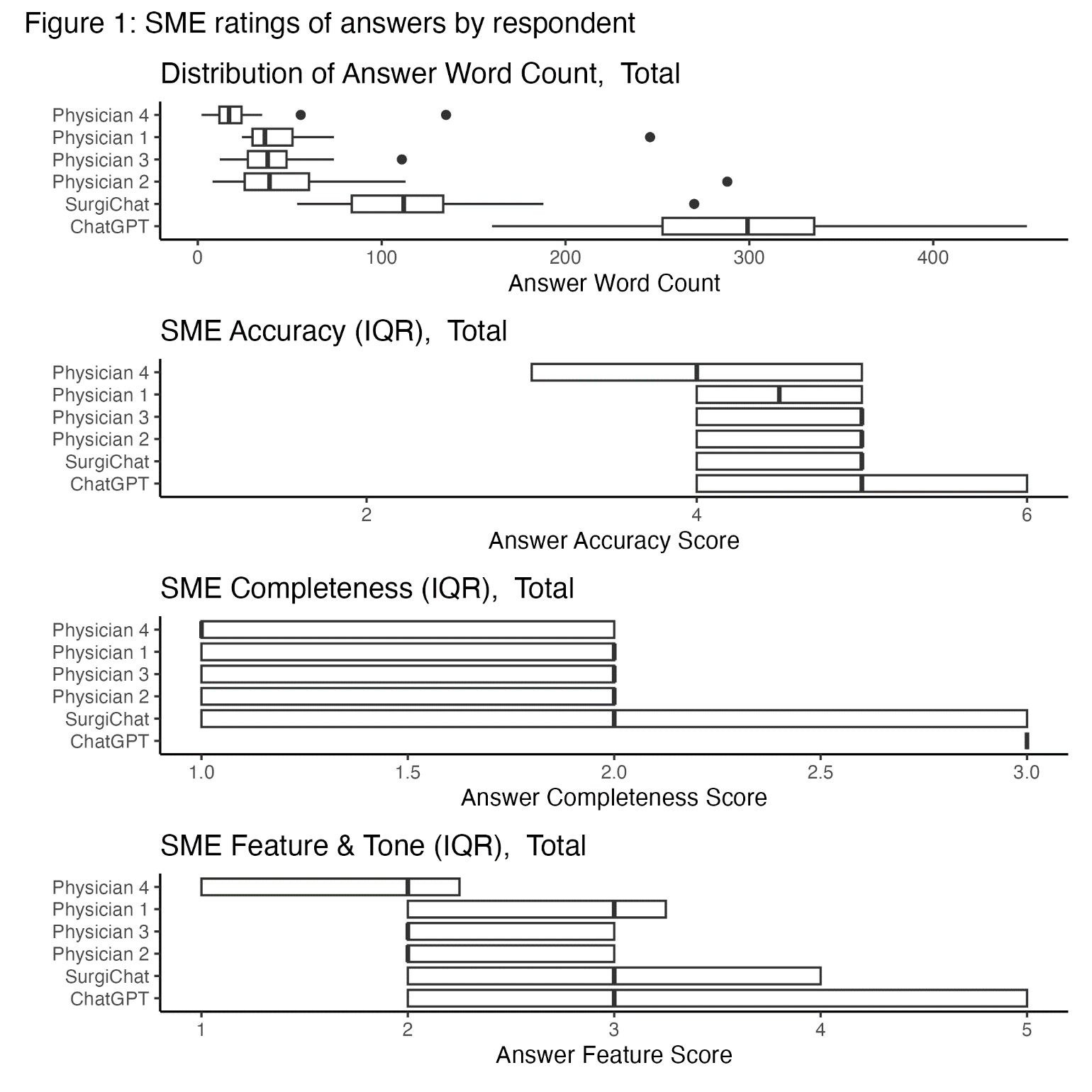


**Supplemental Figure 2: Non-medical volunteer ratings of answers, by respondent**Boxplot of non-medical volunteer evaluations of answers for each respondent with IQR and median, ordered by median word count. Minimum and maximum are omitted from evaluations using Likert scales for clarity.

**
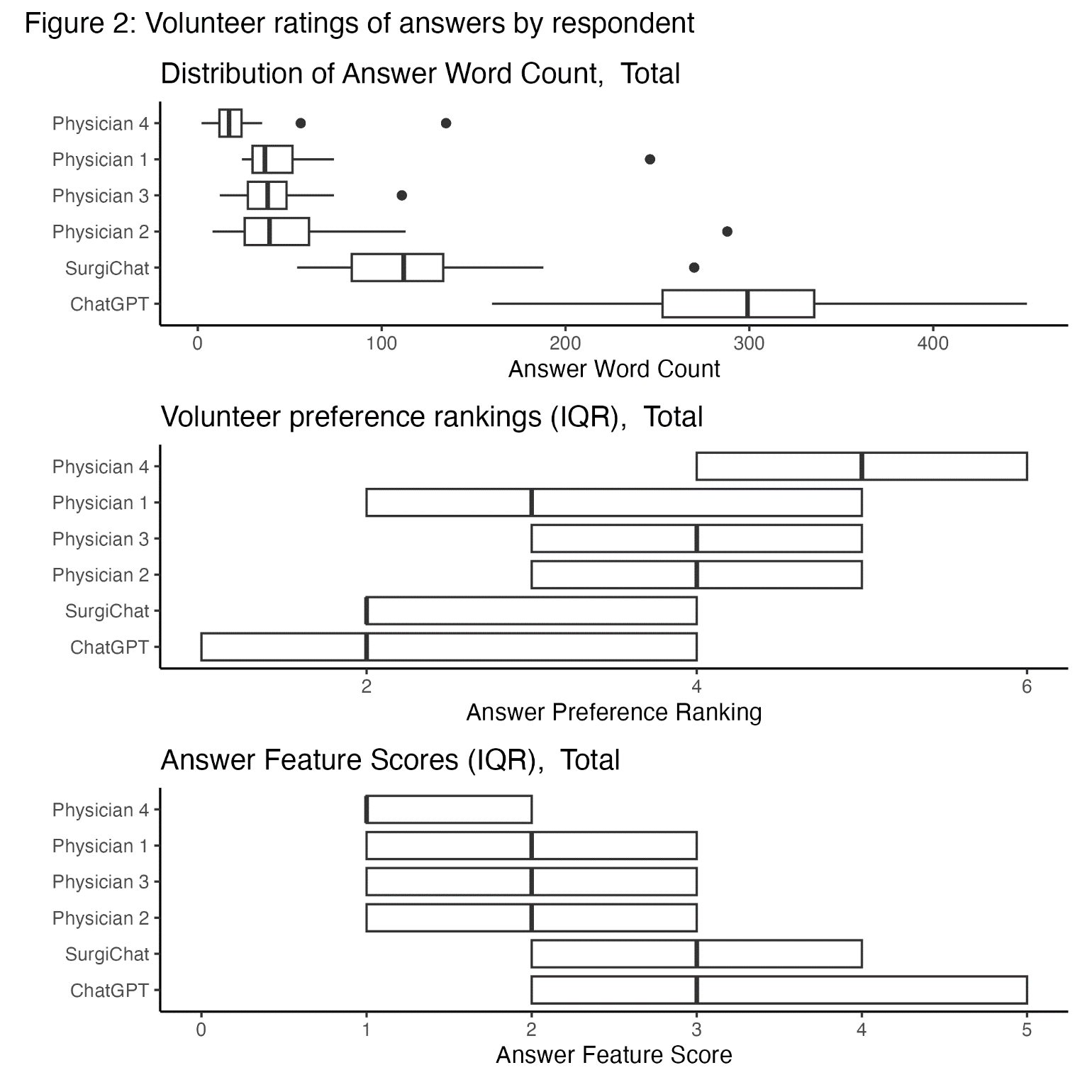
**

**Supplemental Tables**

**Supplemental Table 1: BPH Questions**BPH questions given to chatbots and urologists, drawn from patients in an outpatient urology clinic by the messaging center nursing staff.

|  | Question | Category |
| --- | --- | --- |
| 1 | What is BPH, what are its symptoms and when should I be worried? | Evaluation |
| 2 | Can BPH cause blood in my urine and burning when I urinate? | Evaluation |
| 3 | What is the cause of BPH? Can diet, exercise and supplements help prevent BPH? | Evaluation |
| 4 | What tests are recommended in men with symptoms suggesting BPH? | Evaluation |
| 5 | What are the treatment options for bothersome BPH symptoms beside surgery? | Evaluation |
| 6 | How do I know my problem is not prostate cancer or can progress into cancer? | Evaluation |
| 7 | If BPH is left untreated, what will happen to me? | Evaluation |
| 8 | Which patients should be referred for surgery for BPH? | Operative |
| 9 | TURP, TUVP, Urolift, Rezum, HoLEP: What are the differences between each, and which would be the better option for me? | Operative |
| 10 | Is the TURP procedure dangerous? | Operative |
| 11 | What are the complications of the BPH procedures? | Operative |
| 12 | Do I need blood transfusion for the BPH procedure? | Operative |
| 13 | Do I need anesthesia for the BPH surgery? | Operative |
| 14 | How long do I need to stay in the hospital? | Operative |
| 15 | Am I going to have pain after BPH surgery? | Post-Op |
| 16 | How long does it take to return to my normal activity after BPH surgery? | Post-Op |
| 17 | Will I lose my erections after BPH surgery? Is it safe to take Viagra after my BPH surgery? | Post-Op |
| 18 | Is it normal to see blood after BPH surgery and for how long? | Post-Op |
| 19 | How long do I need to wear my urinary catheter and how to take care of it after BPH surgery? | Post-Op |
| 20 | Will my prostate grow back after surgery for BPH? | Post-Op |

**Supplemental Table 2 Subject Matter Expert evaluations by question category** Subject Matter Expert evaluations of responses on Likert scales for Accuracy (1: completely inaccurate – 6: completely accurate) and Completeness of answer (1: incomplete – 3: comprehensive) as compared to the standardized answer key generated by three separate experts, and on Likert scale for perceived Feature & Tone (1: not empathetic – 5: very empathetic), and stratified by question category.

**
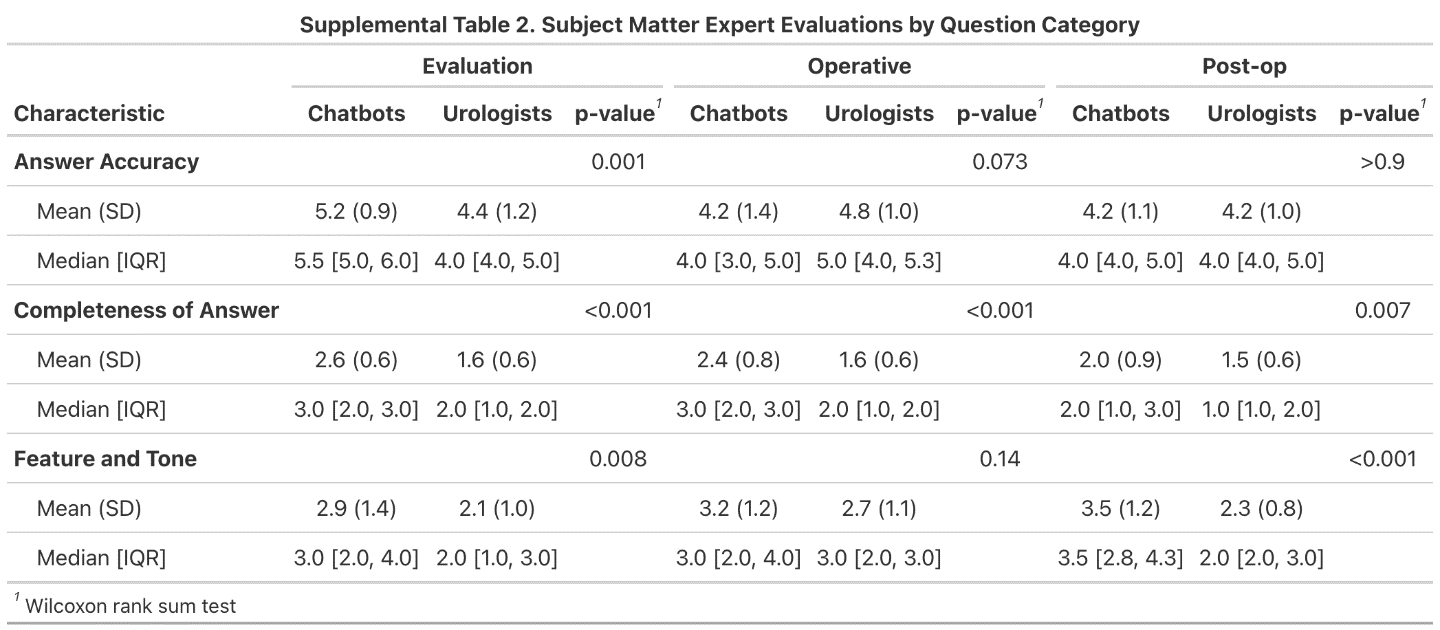
**

**Supplemental Table 3: Non-medical volunteer evaluations based on assumed source**

Non-medical volunteer evaluations of responses, rated on Likert scale for Feature & Tone, (1: not empathetic – 5: very empathetic), ranked preference to receive an answer compared to answers for the same question (1: would most prefer – 6: would least prefer), and accuracy of inferred authorship for each given answer, and stratified by answer category


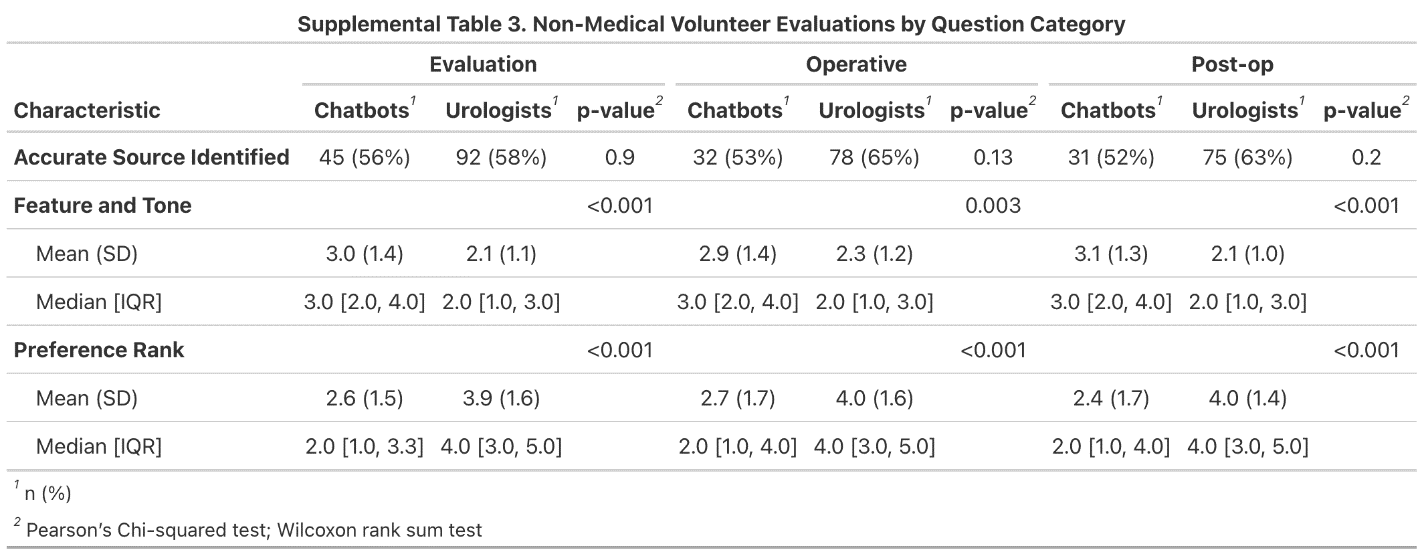


**Supplemental Table 4: Non-medical volunteer evaluations based on assumed source**Non-medical volunteer evaluations of responses based on their inferred authorship for a given response, rated on Likert scale for Feature & Tone, (1: not empathetic – 5: very empathetic), ranked preference to receive an answer compared to answers for the same question (1: would most prefer – 6: would least prefer), and accuracy of inferred authorship for each given answer, and stratified by answer category


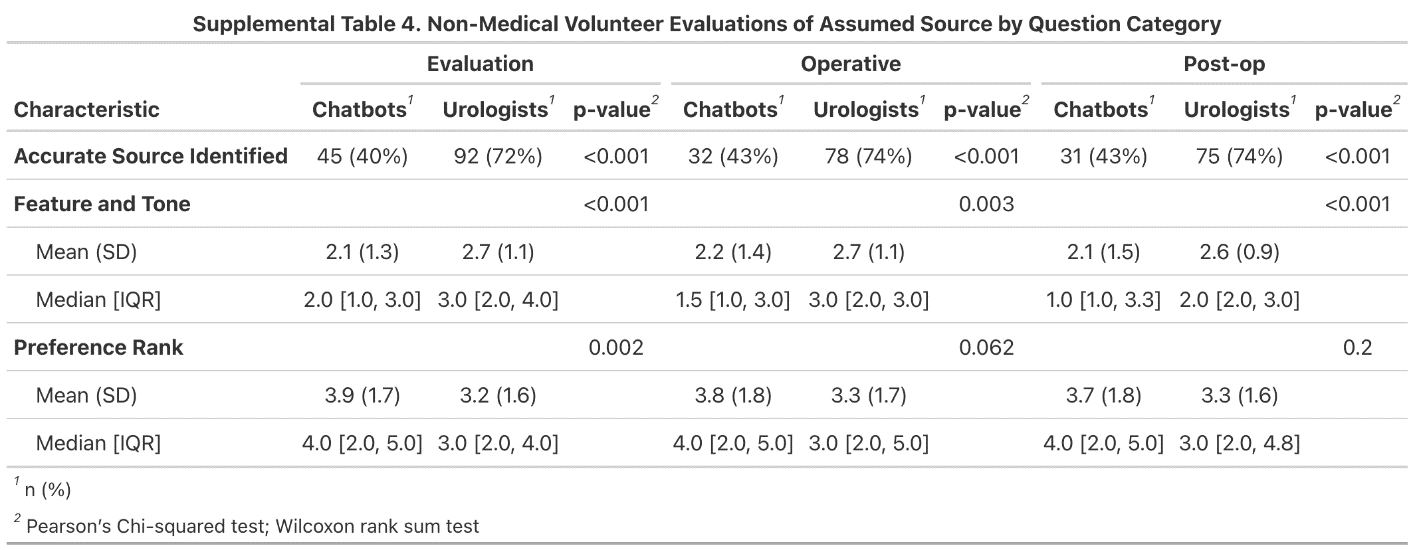

Supplement: Supplementary file 1 — Supplementary Material 1 [file 345_2024_5399_MOESM1_ESM.docx]
